# Internet addiction and functional brain networks: task-related fMRI study

**DOI:** 10.1038/s41598-019-52296-1

**Published:** 2019-10-31

**Authors:** Gergely Darnai, Gábor Perlaki, András N. Zsidó, Orsolya Inhóf, Gergely Orsi, Réka Horváth, Szilvia Anett Nagy, Beatrix Lábadi, Dalma Tényi, Norbert Kovács, Tamás Dóczi, Zsolt Demetrovics, József Janszky

**Affiliations:** 1 Institute of Psychology, University of Pécs, Pécs, Hungary; 2 Department of Neurology, University of Pécs, Medical School, Pécs, Hungary; 3 MTA-PTE Clinical Neuroscience MR Research Group, Pécs, Hungary; 4 Pécs Diagnostic Centre, Pécs, Hungary; 5 Department of Neurosurgery, University of Pécs, Medical School, Pécs, Hungary; 6 MTA-PTE Stress Neurobiology Research Group, Pécs, Hungary; 7 Institute of Psychology, Eötvös Loránd University, Budapest, Hungary

**Keywords:** Addiction, Neural circuits

## Abstract

A common brain-related feature of addictions is the altered function of higher-order brain networks. Growing evidence suggests that Internet-related addictions are also associated with breakdown of functional brain networks. Taking into consideration the limited number of studies used in previous studies in Internet addiction (IA), our aim was to investigate the functional correlates of IA in the default mode network (DMN) and in the inhibitory control network (ICN). To observe these relationships, task-related fMRI responses to verbal Stroop and non-verbal Stroop-like tasks were measured in 60 healthy university students. The Problematic Internet Use Questionnaire (PIUQ) was used to assess IA. We found significant deactivations in areas related to the DMN (precuneus, posterior cingulate gyrus) and these areas were negatively correlated with PIUQ during incongruent stimuli. In Stroop task the incongruent_minus_congruent contrast showed positive correlation with PIUQ in areas related to the ICN (left inferior frontal gyrus, left frontal pole, left central opercular, left frontal opercular, left frontal orbital and left insular cortex). Altered DMN might explain some comorbid symptoms and might predict treatment outcomes, while altered ICN may be the reason for having difficulties in stopping and controlling overuse.

## Introduction

Internet – along with new technologies – has improved many aspects of our lives and it is now essential part of the everyday routine, including professional and social functioning. The benefits that Internet brought into our life are multiple, however, excessive use can contribute to various psychological and medical problems, such as depression^1^, anxiety^2^, body image disturbance^3^, sleeplessness^4^, and poor dietary behavior^3^. Problematic Internet use is considered as a relatively new, fast growing behavioral addiction^5^ that has a potential threat to public health^6^.

Though Internet addiction (IA) is not considered as a distinct mental disorder a more specific form, problematic video gaming, operationalized as ‘Internet gaming disorder’ (IGD), was included in Section 3 (‘Emerging Measures and Models’) of DSM-5, as a condition warranting further study^7^. It is important to note that the 11th revision of the International Classification of Diseases (ICD-11) also includes gaming disorder in section “Disorders Due to Substance Use or Addictive Behaviours”^8^. Several authors claim that it is important to distinguish between IA and IGD. E.g. Montag and colleagues investigated general Internet addiction and specific forms of Internet addiction (incl. video gaming, shopping, social network and pornography) in three European and one Asian country. They found that only online social network showed constant correlation patterns with generalized IA, other specific forms must be distinguished from IA during scientific investigations^9^. Over the last few years, researchers have increased efforts to investigate brain-related alterations to understand the phenomena deeper. These neuroimaging studies – utilizing mainly structural and functional magnetic resonance imaging (MRI) – identified abnormalities in frontal brain regions (orbitofrontal and prefrontal cortex) and the brain’s reward system (putamen, nucleus accumbens) that play crucial role in associative learning^10^ and cognitive control^11,12^. Moreover, IA shares several aspects of substance addiction, such as obsessive thinking about the substance/Internet (daydreaming, rumination, and fantasizing), neglecting everyday activities, social life and essential needs and difficulties in controlling the use^5,13^.

Another common brain-related feature of substance and behavioral addictions is the altered function of higher-order brain networks. Growing evidence suggests that addictions are not only associated with structural and functional breakdown of isolated regions but rather with system-level alterations between brain regions^14^. Functional brain imaging data have revealed that the human brain is topologically organized into a set of coherent spatio-temporal networks, such as default-mode network (DMN)^15^. The DMN was first mentioned by Shulman in 1997^16^ who noted several brain areas in the cerebral cortex that constantly decreased their activity while performing highly demanding tasks. It can be divided into two main subdivisions: the medial prefrontal cortex, and the posterior cingulate cortex with the nearby precuneus and lateral parietal cortex^17^. The DMN-related functional MRI (fMRI) studies can be divided into two types of design. In the resting-state experiments, the subjects lie passively (with closed eyes or with eyes focusing on a fixation cross) without any tasks during scanning. In task-related experiments, fMRI data are acquired during a certain cognitive task and researchers usually focus on deactivations in the brain^18^. However, there are some studies that revealed task-induced activations in the DMN, e.g. when internally directed/self-related cognition is required^19^.

Several studies revealed altered DMN in addictions. These researches focused primarily on gambling disorder^20^ and Internet gaming disorder^15,20^ or substance addictions, such as heroin^21^, alcohol^22^, nicotine^23^, and cannabis^24^. It was also demonstrated that functional connectivity within DMN may predict successful quitting^25^, the intensity of withdrawal-induced craving^26^ and the degree of cognitive decline^27^ in addictions. These observations suggest that dysfunctions in the DMN play important role in the pathogenesis and persistence of addictive disorders. To our knowledge the only study focusing on DMN in adult Internet addicts was conducted by Li and colleagues^28^. They assessed grey matter volumetry and functional connectivity to investigate brain alterations in healthy young adults with an IA tendency. They found altered relationship between the dorsolateral prefrontal cortex (as key node of the cognitive control network) and the anterior cingulate/prefrontal cortices (as key nodes of the DMN).

Impaired inhibitory control is another important feature of addictions (including IA)^29,30^. Pre-existing inhibitory control problems may increase vulnerability to develop addictive disorders, and may serve as a risk factor for their maintenance^31^. Dong *et al*. tried to identify neural correlates of response inhibition in IA. They used Stroop-related fMRI^30^ and event-related brain potential^29^ techniques and showed that cingulate and medial frontal cortices are impaired in IA. There are some practical and theoretical differences in our study, compared with that one conducted by Dong and colleagues. Firstly, they investigated only males with relatively low sample size (24 participants in total). Secondly, we claim, that in the lack of well-established diagnostic criteria and clear cutoff points, continuous measure of IA is recommended. Thirdly, since Stroop task is considered as a highly demanding task, in our study we focused on DMN activation as well (using different contrast in the higher level analysis). Congdon *et al*.^32^ suggested that brain areas related to response inhibition ability should be considered as elements of the “inhibitory control network” (ICN). According to their study, the inferior frontal and medial frontal gyri, the opercular, insular, orbital posterior cingulate and posterior parietal cortices are involved in the ICN.

Taking into consideration the limited number of studies and methods used in previous functional studies in IA, our aim was to investigate the functional correlates of IA in the DMN and in the ICN. To observe these relationships, task-related fMRI responses to verbal Stroop and non-verbal Stroop-like tasks were investigated. We hypothesized that blood-oxygen-level dependent (BOLD) signal changes in the regions of the DMN and ICN are correlated with IA scores. In the lack of well-established diagnostic criteria, we decided to use a multidimensional continuous measure of IA.

## Materials and Methods

### Participants

**Figure 1:**
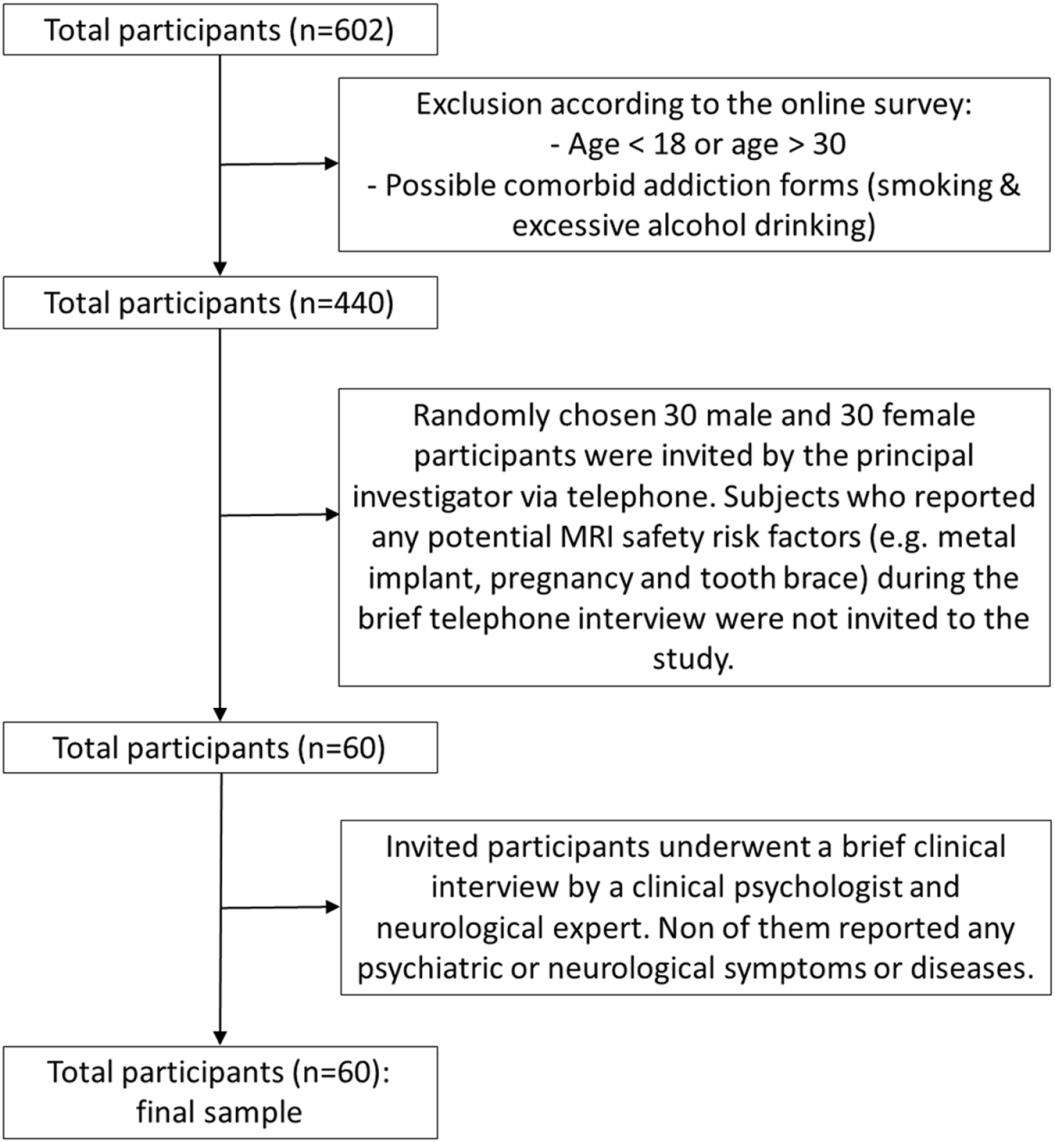
The selection of the final sample. 602 subjects completed the online survey. 139 of them reported smoking habits or excessive alcohol drinking and 28 individuals did not fall within the expected age range (18–30). Since there was overlap between the groups (5 subjects), totally 162 subjects were excluded at this stage of the process. Finally, 30 male and 30 female participants with no neurological and psychiatric symptoms and no risk factors related to MRI measurements were chosen randomly from the remaining 440 subjects.

Our study was conducted through online recruiting. A total of 602 adults participated in the online survey on problematic internet use. According to gender, age and MRI safety parameters sixty healthy Caucasian university students (30 males) aged between 18 and 30 (mean ± SD: 22.0 ± 2.08 years) were included. All participants underwent a brief interview by a clinical psychologist and neurological expert to screen out participants with a current psychiatric and neurologic diagnosis. Subjects with chronic illnesses, neurological or psychiatric disorders were not included (for more details about the selection procedure, see the Fig. 1). All subjects had right-hand dominance according to the Edinburgh Handedness Inventory (Oldfield, 1971). Participants spend on average 2.75 (SD = 2.74) hours online per day and consider themselves 45.27% (SD = 29.05) addicted to the Internet. They were either paid or received course credits for their participation and were naive with regard to the purpose of the experiment.

The study was approved by the Regional Research Ethics Committee of the Clinical Center, University of Pécs.

All procedures performed in studies involving human participants were in accordance with the ethical standards of the institutional and national research committee and with the 1964 Helsinki declaration and its later amendments or comparable ethical standards.

Informed consent was obtained from all individual participants included in the study.

### Assessment

Without clear diagnostic criteria, it is highly recommended to measure excessive Internet use with a continuous questionnaire without using unclear cut-off scores^11^. Therefore, we used the Problematic Internet Use Questionnaire (PIUQ), a validated self-report scale with good reliability and validity characteristics^13,33^. The questionnaire contains 18 items, each scored on a 5-point Likert-type scale ranging from 1 (never) to 5 (always). A confirmatory factor analysis verified the three factor model of questionnaire, each subscale contains six items. Obsession subscale refers to obsessive thinking about the Internet (daydreaming, rumination and fantasizing) and with- drawal symptoms caused by the lack of Internet use (anxiety, depression). (“How often do you feel tense, irritated, or stressed if you cannot use the Internet for as long as you want to?”). Neglect subscale contains items about neglecting everyday activities, social life and essential needs (“How often do you spend time online when you’d rather sleep?”). Control disorder subscale reflects difficulties in controlling time spent on the Internet (“How often do you realize saying when you are online, ‘just a couple of more minutes and I will stop’?”). Since in this study we focused on global psychological consequences of Internet addiction, we used PIUQ total score in statistical analyses, that was computed by summing the scores on all the items of the scale.

### Stimuli

Using Presentation software (Neurobehavioral Systems, Inc., Berkeley, CA, USA), the visual stimuli were presented via MRI-compatible goggles (VisualSystem, NordicNeuroLab AS, Bergen, Norway) and subjects’ responses were collected via MRI-compatible response buttons (ResponseGrip, NordicNeuroLab AS, Bergen, Norway).

Since indirect behavioral evidences suggest that language network and verbal processes might be impaired in IA^34^, we decided to use two different tasks in this study for controlling this possible interfering effect: verbal Stroop task and non-verbal Stroop-like task.

### Verbal stroop task

A series of colored words were displayed against a black background. Half of the words were written in the same ink color as the meaning of the word (congruent stimuli, e.g., the word “green” displayed in green color), while the other half were written in colors other than the word’s meaning (incongruent stimuli, e.g., the word “green” displayed in blue color). Four colored words (blue, green, red, and yellow) and their corresponding colors were used. Subjects had to choose the ink color (and neglect the meaning) of the words by using the four response buttons (left thumb = red; left index = blue; right thumb = green; right index = yellow). Colored circle thumbnails were presented on the bottom of screen to aid the subjects in which button to use for different colors (Fig. 1).

### Non-verbal stroop-like task

A series of white arrows pointing either left or right was displayed against a black background either on the left or right side of a centered fixation cross. Half of the stimuli were pointing in the same direction as their position on the screen (congruent stimuli, e.g., a leftward pointing arrow on the left side of the fixation cross), while the other half were pointing in an opposite direction as their position on the screen (incongruent stimuli, e.g., leftward pointing arrow on the right side of the fixation cross). Subjects had to choose the direction (and neglect the position) of the arrows by pressing one of the two buttons with their left or right index fingers.

### Experimental design

Stimulus presentations were analogous in both tasks. Stimuli were presented for 1200 ms each, with an interstimulus duration of 800 ms. Sixteen stimuli were presented in each session (32 s), with 8 congruent and 8 incongruent stimuli, presented pseudo randomly. Half of the stimuli required left-handed, while the other half required right-handed responses within each session.

**Figure 2:**
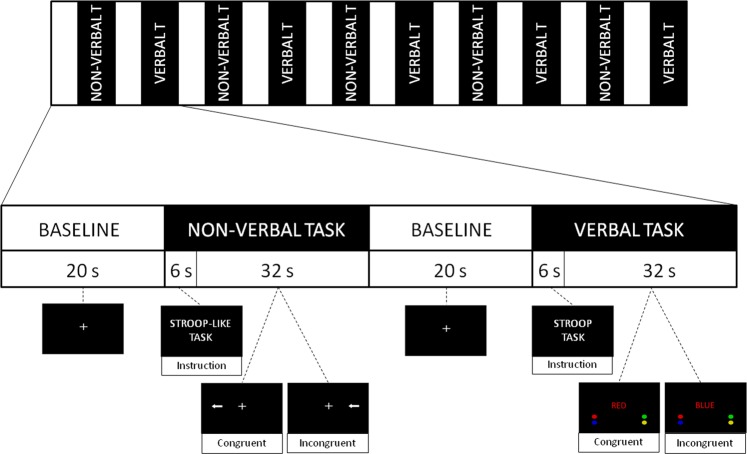
Verbal Stroop and Non-verbal Stroop-like task: design and stimuli examples. The experiment started with a baseline session. It was followed by task sessions that were presented successively. Subjects had to choose the direction of the arrows (Stroop-like task) or ink color of the words (Stroop task).

The experiment started with a 20 s baseline session with a fixation cross in the center of the screen. The baseline section was followed by the first nonverbal Stroop-like task session that started with a 6 s instruction period followed by the congruent and incongruent stimuli. Then, a 20 s baseline session with fixation cross was conducted following by the verbal Stroop task (again, starting with 6 s instruction period). The verbal Stroop and non-verbal Stroop-like task sessions were presented successively, always interleaved with 20 s baseline. The whole experiment consisted of 5–5 repetitions with 10 baseline sessions, resulting in a total measurement time of 580 s (Fig. 2). Reaction times (RTs) and error rates (ERs) were recorded for each condition (verbal_congruent, verbal_incongruent, non-verbal_congruent, non-verbal_incongruent).

### Imaging data acquisition and visual analysis

All measurements were performed on a 3 T Magnetom TIM Trio human whole-body MRI scanner (Siemens AG, Erlangen, Germany) with a 12-channel head coil. Functional images were acquired using a 2D single-shot echo-planar imaging (EPI) sequence (TR/TE = 2000/30 ms; Flip angle = 76°; 36 axial slices with a thickness of 3 mm; FOV = 192 × 192 mm^2^; matrix size = 64 × 64; receiver bandwidth = 2170 Hz/pixel; no gap; interleaved slice order to avoid crosstalk between contiguous slices). For distortion correction purposes, field mapping sequence (TR/TE1/TE2 = 400/4.92/7.38 ms; Flip angle = 60°; 36 axial slices; FOV = 228 × 228 mm^2^; matrix size = 76 × 76; receiver bandwidth = 259 Hz/pixel) with the same voxel size, orientation and adjustment parameters as the fMRI scan was acquired right after the fMRI measurement. Anatomical images were obtained using an isotropic T1-weighted 3D-MPRAGE sequence (TR/TI/TE: 2530/1100/3.37 ms; Flip angle = 7°; 176 sagittal slices with a thickness of 1 mm; FOV = 256 × 256 mm^2^; matrix size = 256 × 256; receiver bandwidth = 200 Hz/pixel). The MPRAGE anatomical images were visually checked by MRI experts. There were no brain abnormalities according to the visual analysis of the MR images.

### Functional MRI data processing and analysis

Pre-processing and statistical analyses were performed using FEAT (FMRI Expert Analysis Tool) Version 6.00, part of FSL (FMRIB’s Software Library, http://www.fmrib.ox.ac.uk/fsl). Pre-processing included MCFLIRT motion correction, slice timing correction, brain extraction, spatial smoothing with 5 mm full width at half maximum, and a high-pass temporal filter of 104 s (estimated using FSL’s cutoffcalc tool). The temporal filtering applied to the data was used for the model as well. Whole brain general linear model (GLM) time-series statistical analyses of individual data sets were carried out using FILM (FMRIB’s Improved Linear Model) with local autocorrelation correction.

First level analysis included five regressors: verbal (Stroop task) congruent, verbal incongruent, non-verbal (Stroop-like) congruent and non-verbal incongruent conditions, and an additional regressor to model BOLD changes induced by the task instructions.

The single-session data sets were registered into the MNI152 standard space using a two-step process. First, functional (EPI) image of each subject was registered to that subject’s T1 structural scan using BBR (6 degrees-of-freedom) incorporating simultaneous B0 field unwarping (i.e. distortion correction) with a combination of FUGUE and BBR tools^35,36^. Then, each subject’s T1 image was registered to the 2 mm MNI152 standard space T1 image using a 12 degrees-of-freedom linear fit followed by nonlinear registration (FNIRT, warp resolution = 8 mm). Next, for each subject, these two registrations were combined and applied to the first-level statistical maps to take them into standard space.

Second-level mixed-effects analyses were carried out using FLAME (FMRIB’s Local Analysis of Mixed Effects, stage 1 and 2) with outlier de-weighting to investigate the following questions:

i. Deactivation pattern during high-demand incongruent condition (verbal and non-verbal separately).
ii. Correlation between PIUQ and BOLD signal change during high-demand incongruent condition (verbal and non-verbal tasks separately).
iii. Activation pattern during the incongruent condition (verbal and non-verbal separately).
iv. Correlation of PIUQ with BOLD signal change between incongruent and congruent conditions (incongruent_minus_congruent; verbal and non-verbal separately).

Statistical maps were considered to be significant at Z > 2.3 and a family-wise error corrected cluster significance threshold of p = 0.05.

Brain areas were considered to be part of the DMN if the following assumptions were satisfied for the local maximas (LMs): a) deactivation measured during incongruent condition and b) according to the Harvard-Oxford Cortical Structural Atlas (https://fsl.fmrib.ox.ac.uk/fsl/fslwiki/Atlases) the LMs are in the areas related to the DMN (incl. medial prefrontal, posterior cingulate cortex, precuneus or lateral parietal cortex)^17^.

Brain areas were considered to be part of the ICN if the following assumptions were satisfied for the LMs: a) activation measured during incongruent_minus_congruent contrast indicating congruency effect or interference effect; b) according to the Harvard-Oxford Cortical Structural Atlas the LMs are in the areas related to the ICN (incl. inferior frontal and medial frontal gyri, the opercular, insular, orbitofrontal, posterior cingulate and posterior parietal cortices)^32^.

### Statistical analyses for behavioral data

Statistical analyses were performed using IBM SPSS Statistics for Windows, Version 22.0 (IBM Corp. Released 2013. Armonk, NY, USA). Mean reaction time (RT) and error rate (ER) differences between congruent and incongruent stimuli were assessed by paired samples t-test or Wilcoxon signed-rank test depending on the distribution of the data. Since PIUQ scores did not show normal distribution, Spearman’s rank correlation was used to study the associations between RTs/ERs and PIUQ.

## Results

### Behavioral results

The mean of the total score on the PIUQ for our sample is 32.85 (SD = 12.4, 95% CI: [29.62–36.08].

Significant differences were found between congruent and incongruent stimuli in RTs and ERs regarding both the verbal Stroop and the non-verbal Stroop-like task (Table 1). No significant correlations were found between task performance scores (incl. reaction times – RT, error rates – ER and congruency effect [incongruent_minus_congruent for RTs and ERs) and PIUQ total.

**Table 1:** Differences in task performances between conditions and correlations with PIUQ.

Task	Condition	Mean (SD) or Median (min-max)	t/Z	rho
**Correlation with PIUQ**				
Verbal Stroop RT^a^	Cong.	0.87 (0.09)	4.96***	0.077
	Incong.	0.96 (0.17)		0.077
Verbal Stroop ER^b^	Cong.	1 (0–18)	5.80***	−0.01
	Incong.	5 (0–18)		−0.02
Non-verbal Stroop RT^a^	Cong.	0.65 (0.10)	8.43***	−0.11
	Incong.	0.68 (0.10)		−0.12
Non-verbal Stroop ER^b^	Cong.	0 (0–4)	4.16***	0.15
	Incong.	1 (0–5)		0.04

^a^Paired samples t-test, mean (SD) and t values are presented;

^b^Wilcoxon signed-rank test, median (min-max) and Z scores are presented;

***p < 0.001, RT – reaction time, ER – error rate, PIUQ – Problematic Internet Use Questionnaire, rho – Spearman’s correlation coefficient.

### fMRI results

**Figure 3:**
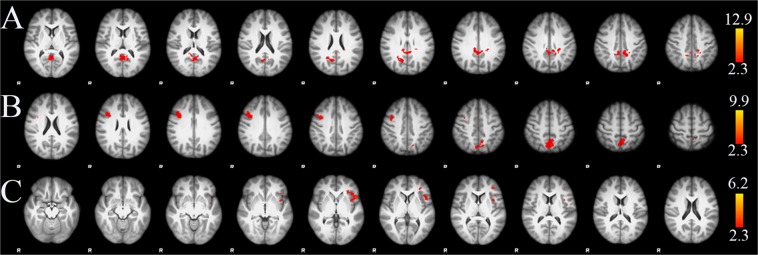
Group level negative associations between PIUQ and BOLD signal changes during incongruent condition in verbal Stroop task (**A**) and non-verbal Stroop-like task (**B**). Group level positive associations between PIUQ and BOLD signal changes during incongruent_minus_congruent contrast in Stroop task (**C**). Images were thresholded using clusters determined by Z > 2.3 and a corrected cluster significance threshold of p = 0.05. Axial slices are shown in radiological convention for MNI slice coordinates from Z = 8 to 48 mm in (**A**), Z = 22 to 62 mm in (**B**) and Z = −16 to 24 mm in (**C**).

The correlations between PIUQ and BOLD fMRI results for incongruent and incongruent_minus_congruent contrasts are presented in Table 2 (see also Fig. 3). During the incongruent stimuli in the verbal Stroop task, significant negative correlations were found in the bilateral posterior cingulate gyri and bilateral precuneus. During the incongruent stimuli in non-verbal Stroop-like task, significant negative correlations were detected in the bilateral precuneus, the right middle frontal gyrus and right precentral gyrus. BOLD signal was decreased with the severity of Internet addiction in both tasks.

**Table 2:** Correlations between PIUQ and fMRI BOLD signal changes.

Cluster	Area	#voxels	Max. Z-score	MNI coordinates		
				x	y	z
**PIUQ INCONGRUENT**						
	*Verbal Stroop task*					
1.	Left posterior cingulate gyrus^a^	506	4.09	0	−42	34
	Right precuneus^a^			14	−50	46
	Right posterior cingulate gyrus^a^	434	3.78	8	−40	30
2.	Left precuneus^a^			−4	−58	10
	*Non-verbal Stroop-like task*					
1.	Left precuneus^a^	386	3.95	−2	−60	52
	Right precuneus^a^			8	−68	50
2.	Right middle frontal gyrus^a^	378	4	40	8	38
	Right precentral gyrus^a^			42	2	42
**PIUQ INCONGRUENT_MINUS_CONGRUENT**						
	*Verbal Stroop task*					
1.	Left inferior frontal gyrus pars opercularis^b^	275	3.45	−50	16	0
	Left frontal pole^b^			−36	40	8
	Left central opercular cortex^b^			−38	8	10
	Left frontal opercular cortex^b^			−42	24	2
	Left frontal orbital cortex^b^			−44	20	−6
	Left insular cortex^b^			−42	10	−2
	*Non-verbal Stroop-like task*	n.s.				

^a^Negative correlation ^b^positive correlation; n.s. not significant; MNI coordinates are listed for the local maximas.

Incongruent_minus_congruent contrast for Stroop task positively correlated with PIUQ in several left sided cortical areas including the inferior frontal gyrus (pars opercularis), frontal pole, central and frontal opercular cortex, frontal orbital cortex and insular cortex. The same contrast for non-verbal Stroop-like task showed no significant relationship with PIUQ.

Compared to the baseline, significant deactivations were found in the areas related to the DMN during incongruent stimuli in Stroop and Stroop-like tasks (see Fig. S1). Areas related to the ICN showed significant activation during incongruent stimuli in Stroop task (Fig. S2).

## Discussion

In this study, functional correlates of Internet addiction were demonstrated during verbal Stroop and non-verbal Stroop-like task in young adult Internet users. Since the different task-related fMRI contrasts represent different psychological and neural domains they will be discussed separately.

Incongruent stimuli induced BOLD signal changes were found to be negatively correlated with PIUQ in bilateral precuneus and posterior cingulate gyri (PCG) during Stroop task and bilateral precuneus, during Stroop-like task. Moreover, we found significant deactivations in these areas during incongruent stimuli for both verbal Stroop and non-verbal Stroop-like tasks. Brain areas in the cerebral cortex with constantly decreasing activity during demanding tasks are considered being part of the DMN^16^. Previous studies investigating Internet addiction and Internet gaming disorder (IGD) also found alterations in the DMN. However, these studies revealed the involvement of the anterior part of the network and used merely resting-state fMRI. Li *et al*.^28^ demonstrated decreased anticorrelation between the right dorsolateral prefrontal cortex and the anterior part of the DMN (medial prefrontal cortex and anterior cingulate gyrus) that might lead to dysfunctions of the cognitive control network and DMN, including diminished cognitive efficiency. Wang *et al*. also revealed reduced functional connectivity in the anterior part of the DMN, as well as decreased interactions between the salient network and DMN in adolescents^37^. Authors claim that this may serve as neural background of the uncontrolled Internet use and they also suggest that IA may share similar neurobiological abnormalities than other addictions. Similar impaired connectivity patterns were found in IGD^15^. In addition, functional connectivity among DMN regions were in negative correlation with anger and depression^38^, as well as with executive control dysfunction^39^. The results of our study have significant contribution to advance our knowledge on the field. We are the first to show that task-related changes in activation of DMN-related structures are also significantly related to IA. The highlighted areas (precuneus and PCG) are parts of the posterior part of the DMN and previous studies revealed that the middle frontal gyrus observed in the current study is also strongly related to the DMN^40^.

Impaired DMN may explain some comorbid symptoms that often occur in IA, thereby it may have important clinical implications. Firstly, many evidences indicate that DMN plays an important role in cognition, particularly in attention and memory processes. Wide range of studies showed that vigilance^41^, semantic processing^42^, episodic, autobiographical memory^43^ and creative problem solving^44^ are strongly associated with DMN activation, moreover, diminished vascular functionality for the DMN in mid-life may serve as a preclinical marker for brain dysfunction in later life^45^. These observations may implicate long term negative effect of IA on cognition - similarly to those presented in nicotine addiction^27^, and suggest that DMN alterations serve as the common neuro-functional background behind IA and attention-related deficits^46^. Although, it must be note that IA is still a relatively young phenomenon and more evidences are needed to prove this assumption. Secondly, according to previous studies connectivity within DMN can be changed with pharmacotherapies via bottom-up and with cognitive behavioral therapy (CBT) via top-down mechanisms in some psychiatric disorders (for review see^47^), however, the exact mechanisms are unclear. Furthermore, individual variations in pre-treatment functional connectivity may be a reliable predictor of treatment efficacy in schizophrenia^48^, major depression^49^ and smoking^27^. It is important to note that these studies did not investigate the DMN as a holistic functional brain system, they focused rather on its connection with other networks (executive control network)^27^, on a highlighted component of the DMN (dorsolateral prefrontal cortex)^48^ and subnetworks of the DMN (anterior and posterior subnetworks; abnormal functional connectivity within the posterior part was normalized after antidepressant treatment, while anterior subnetwork remained persistent against treatment)^49^. Since CBT seems to be successful methods for improving IA status^50^, individual differences in DMN might also predict treatment outcome and be reliable and objective tool for assessing therapeutic efficacy. Further studies are needed to explore this.

In Stroop task the incongruent minus congruent contrast showed significant positive correlation with PIUQ in the left inferior frontal gyrus, left frontal pole, left central opercular, left frontal opercular, left frontal orbital and left insular cortex. Activation in these areas were reported to be correlated with individual differences in task performances requiring good inhibition ability^32,51^. Congdon *et al*. used Stop-signal task-related probabilistic ICA (independent component analysis) and referred two components that included similar regions found in our study^32^. They demonstrated that engagement of networks that include these regions is positively related to response inhibition that is the ability to suppress a habitual response or routine behavior, including motor actions and higher-order responses (thoughts, emotions etc.). Therefore, these networks are essential to stop problematic behaviors – such as excessive Internet use. Ergo, it is not surprising that Internet addiction was found to be associated with impaired inhibition in previous studies^30,52^. Here we suggest that a special “inhibitory control network (ICN)” that was found by Congdon and colleagues^32^ should be considered as a possible pathogenic factor in the development of IA. However, significant correlations were only found during the Stroop task as an important unexpected result. The most plausible explanation is that the Stroop-like non-verbal task was not sensitive enough to detect individual differences due to the relatively simple nature of the task even in incongruent condition. Despite there were significant differences between incongruent and congruent conditions in RT and ER, incongruency effect did not occur in an expected way. Another possible explanation might be that the verbal domain is altered in IA. This is supported by the presence of left hemisphere specialization in our study that is known to be typical for language processing^53^. At this stage of knowledge, this explanation seems highly speculative, and further studies are needed.

Some limitations must be considered. First, the cross-sectional nature of the study limits our ability to discriminate between cause and effect. Does excessive Internet use lead to brain-related changes or vica versa? Secondly, IA is known to have various comorbid symptoms such as depression, attention deficit hyperactivity disorder, anxiety disorders etc. Although, we excluded patients with diagnosed comorbid diseases, subjects with subclinical symptoms may have influenced our results. Another limitation is related to recruitment through online platforms. The major problems with this method are that the representativeness of the sample cannot be fully determined and problematic Internet users could be overrepresented in the sample.

Taken together, our results suggest that problematic Internet use in healthy young adults has functional brain correlates in regions that are related to the DMN. Since similar associations were found in IGD^54^, smoking^18^ and other substance related addictions^21^ our results suggest long-term negative effects of IA on brain functions. As described before, DMN is strongly related to human cognitive performance and some features of the DMN were able to predict therapy outcomes in psychiatric disorders. Therefore, we suggest, that altered DMN might explain some comorbid symptoms (including attention deficit) and might predict treatment outcomes (for further information about Internet-related changes in human cognition see the review article of Loh & Kanai^55^). Although we must highlight, that in the lack of well-designed, reliable researches, these assumptions are speculative at the moment. Activation in the ICN was also found to be correlated with problematic Internet use suggesting that difficulties in stopping and controlling overuse are not results of “weak character of the person” but the impaired brain mechanisms responsible for behavioral inhibition. However, it must be highlighted that, since we investigated only healthy young people, our conclusions should be applied only to this group. Longitudinal studies and studies with other age groups (e.g. adolescents) are highly needed to test if these findings can be generalized to other groups and whether these functional correlates are the result of overall additive tendencies or specific to maladaptive Internet use. It would be also important to know that controlled Internet use or complete abstinence could reduce these brain-related changes.

## Supplementary information

LaTeX Supplementary File

LaTeX Supplementary File
