# Supplementary material for: Internet addiction and functional brain networks: task-related fMRI study
Source: Sci Rep. 2019 Oct 31;9:15777. doi: 10.1038/s41598-019-52296-1 (PMC6823489; doi:10.1038/s41598-019-52296-1)
Supplement: Supplementary file 2 — LaTeX Supplementary File [file 41598_2019_52296_MOESM2_ESM.docx]

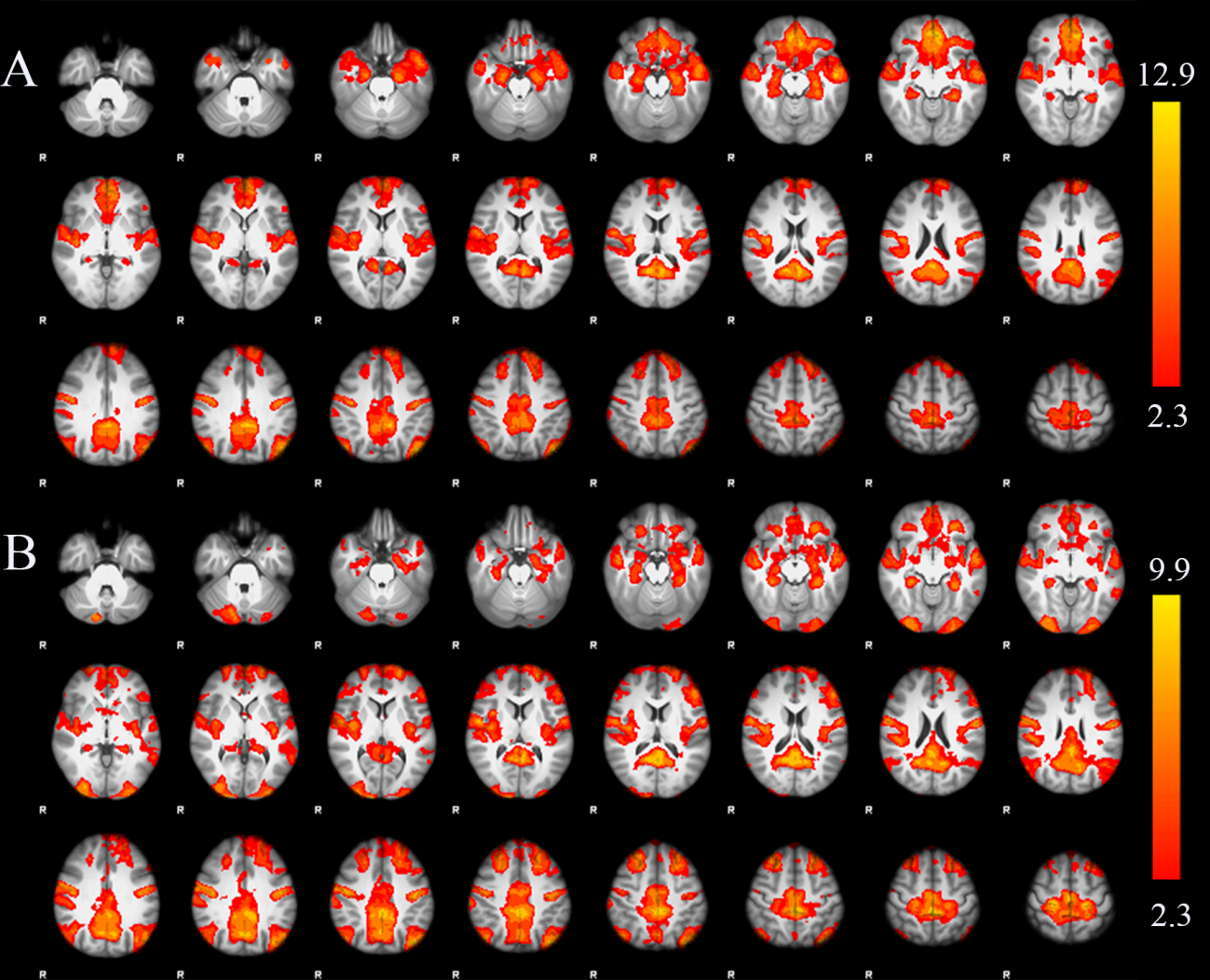


**Supplementary material 2.** Deactivation during incongruent stimuli in A) verbal Stroop task and B) non-verbal Stroop-like task. Images were thresholded using clusters determined by Z > 2.3 and a cluster significance threshold of p = 0.05. Axial slices are shown in radiological convention. The figures are shown in the same coordinates: MNI slice coordinates from Z = -34 to 62 mm.
